# Discrimination, harassment, and intimidation amongst otolaryngology: head and neck surgeons in Canada

**DOI:** 10.1186/s40463-022-00590-w

**Published:** 2022-09-30

**Authors:** Amr F. Hamour, Tanya Chen, Justin Cottrell, Paolo Campisi, Ian J. Witterick, Yvonne Chan

**Affiliations:** Department of Otolaryngology—Head and Neck Surgery, University of Toronto, 6 Queen’s Park Cres. W, Toronto, ON M5S 3H2 Canada

**Keywords:** Otolaryngology—head and neck surgery, Mistreatment, Discrimination, Gender, Diversity, Canada

## Abstract

**Background:**

Understanding mistreatment within medicine is an important first step in creating and maintaining a safe and inclusive work environment. The objective of this study was to quantify the prevalence of perceived workplace mistreatment amongst otolaryngology—head and neck surgery (OHNS) faculty and trainees in Canada.

**Methods:**

This national cross-sectional survey was administered to practicing otolaryngologists and residents training in an otolaryngology program in Canada during the 2020–2021 academic year. The prevalence and sources of mistreatment (intimidation, harassment, and discrimination) were ascertained. The availability, awareness, and rate of utilization of institutional resources to address mistreatment were also studied.

**Results:**

The survey was administered to 519 individuals and had an overall response rate of 39.1% (189/519). The respondents included faculty (n = 107; 56.6%) and trainees (n = 82; 43.4%). Mistreatment (intimidation, harassment, or discrimination) was reported in 47.6% of respondents. Of note, harassment was reported at a higher rate in female respondents (57.0%) and White/Caucasian faculty and trainees experienced less discrimination than their non-White colleagues (22.7% vs. 54.5%). The two most common sources of mistreatment were OHNS faculty and patients. Only 14.9% of those experiencing mistreatment sought assistance from institutional resources to address mistreatment. The low utilization rate was primarily attributed to concerns about retribution.

**Interpretation:**

Mistreatment is prevalent amongst Canadian OHNS trainees and faculty. A concerning majority of respondents reporting mistreatment did not access resources due to fear of confidentiality and retribution. Understanding the source and prevalence of mistreatment is the first step to enabling goal-directed initiatives to address this issue and maintain a safe and inclusive working environment.

**Graphical Abstract:**

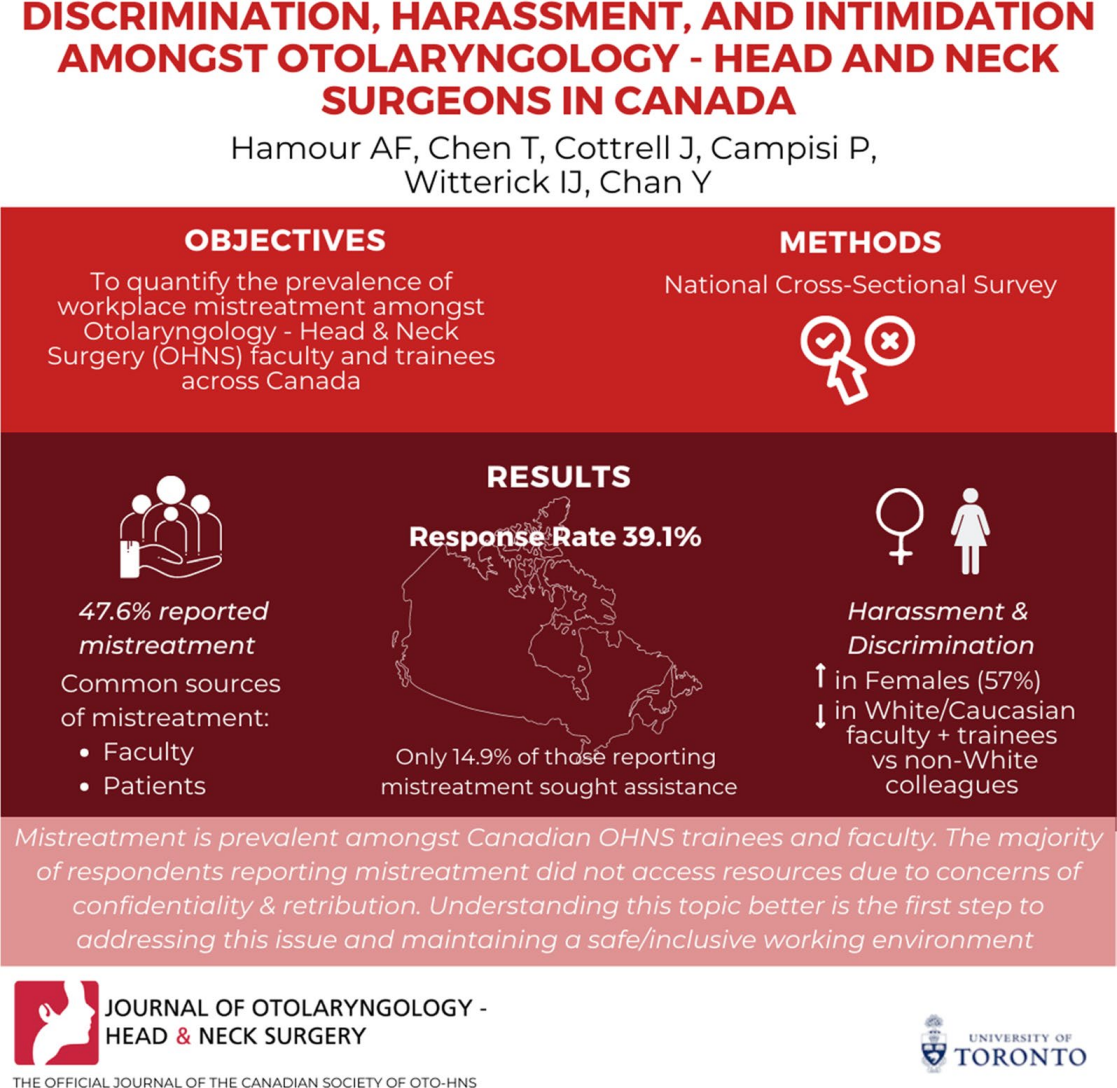

## Background

Physicians and learners in medical and surgical subspecialties often experience mistreatment in their work environment [1–3]. The hierarchical nature of medicine and a lack of diversity are historical factors that have contributed to mistreatment in the clinical setting. The sources of mistreatment (discrimination, verbal abuse, and physical abuse) have extended beyond the faculty-learner relationship and included sources such as interactions with nurses and patients [4, 5]. In fact, several studies have shown that the most commonly cited source of abuse and mistreatment are patients and their families [5, 6]. A recent survey directed at surgical residents in the United States revealed that 66% of female residents and 20% of male residents have experienced gender or race-based discrimination [5]. Another survey amongst female medical faculty and residents in Otolaryngology-Head and Neck Surgery (OHNS) found that only 53% reported a “harassment-free” environment within their workplace [7].

In addition to gender, *visible minority* (VM) status has also been associated with increased levels of mistreatment in medicine. The term *visible minority* is defined by the Canadian government as “persons other than aboriginal peoples, who are non-Caucasian in race or non-White in colour” [8]. A Canadian study by Mocanu and colleagues (2020) reported that general surgery trainees identifying as VM perceived a higher rate of disregard of their medical expertise and that they were more likely to worry about employment opportunities due to their race and ethnicity [9].

There is a paucity of data characterizing the source, nature and prevalence of mistreatment in OHNS in Canada and North America. Therefore, the primary objective of this study was to characterize perceived workplace mistreatment amongst OHNS faculty and trainees in Canada.

## Methods

Ethics approval was obtained from the University of Toronto Health Sciences Research Ethics Board (Protocol #00040225).

### Study design

This was a cross-sectional survey study. There were no incentives offered for completion of the survey and participation was voluntary. The survey (Appendix) was distributed via email and fax to addresses listed on a comprehensive list of all otolaryngologists and otolaryngology resident trainees in Canada. Invitations were sent to 348 practicing otolaryngology faculty and 171 resident physicians. Informed consent to analyze data was obtained from respondents. The invitations included a weblink to the survey. The data collection platform used was SurveyMonkey (SVMK Inc., USA). All respondents had the option to end participation in the survey at any stage and review or edit their responses. Access to the survey was available for a period of four weeks.

### Survey content

Survey question development was an iterative process led by a focus group consisting of three OHNS faculty (PC, IW, YC) and three OHNS trainees (AH, TC, JC). The survey contained 33 questions and was available in both English and French languages. Translation into French was performed by two native language speakers and cross-checked for any sources of bias in order to preserve question intent.

The target population included practicing otolaryngologists and trainees in Canada. ‘Faculty’ were defined as practicing otolaryngologists that were certified by the Royal College of Physicians and Surgeons of Canada or equivalent. ‘Trainees’ were defined as resident physicians currently enrolled in a Royal College accredited training program. Collected demographic information included gender, self-reported ethnicity, and status as trainee or faculty. To preserve anonymity, identifying information such as name, age, and institution affiliation were not requested.

The first section of the survey focused on the prevalence and degree of mistreatment in the workplace experienced over the previous year. Mistreatment was defined as intimidation, harassment, or discrimination in any form and is detailed in Table 1. The definitions were provided to the respondent in the stem of the relevant survey questions.

**Table 1:** Definitions of relevant concepts within the survey

Concept	Definition
Mistreatment	Intimidation, harassment, or discrimination in any form
Intimidation	Any behaviour, educational process, or tradition that induces fear in an individual or has a detrimental effect on the working environment
Harassment	A course of vexatious comment or conduct which the person knows or ought reasonably to know is unwelcome [19]
Discrimination	Unequal treatment based on ancestry, citizenship, colour, disability, ethnic origin, religion/faith/belief system, family status, gender expression, gender identity, marital status, place of origin, race, sex (including pregnancy), and sexual orientation that was direct, indirect, subtle, or overt [20]

The second section of the survey focused on the sources of mistreatment. The options listed in the questions included: patient or patient family member, faculty (OHNS and non-OHNS), nurse, allied health professional (physiotherapist, audiologist, etc.), or trainee (fellow, OHNS resident and non-OHNS resident).

The final section of the survey assessed the presence, awareness, and utilization of institutional policies and resources to address mistreatment. There was a free text option at the end of the survey for additional comments.

### Statistical analysis

Categorical variables were compared using chi-square test. Confidence intervals for the difference in proportions was calculated. Analysis was performed using SPSS® version 26.0 (SPSS, Chicago, IL).

## Results

### Demographics

The survey was administered to 519 individuals and had an overall response rate of 39.1% (189/519). The respondents included faculty (n = 107; 56.6%) and trainees (n = 82; 43.4%). There was a higher response rate amongst trainees compared to faculty (48.0% versus 30.2%). Table 2 summarizes the demographic details of the respondents. The most common self-identified ethnicities were White/Caucasian (n = 112; 59.3%), Middle Eastern (n = 22; 11.6%), and East Asian (n = 19; 10.0%). Mistreatment (intimidation, harassment, or discrimination), as defined in Table 1, was reported by 47.6% (n = 90) of respondents. A greater proportion of trainees reported mistreatment compared to faculty (n = 44; 53.7% versus n = 46; 43.0%).

**Table 2:** Demographic data of survey respondents

	n =	% of total
*Training level*		
Faculty (academic practice)^a^	59	31.4
Faculty (community practice)^b^	48	25.5
Trainee	81	43.1
Resident PGY1-2	35	18.6
Resident PGY3-5	46	24.5
*Years in practice (faculty)*		
Less than 5	21	19.8
6–10	23	21.7
11–20	35	33.0
21–29	18	17.0
More than 30	9	8.5
*Gender*^*c*^		
Male	103	56.9
Female	77	42.5
Not sure/questioning	1	0.6
*Self-identification*		
White/Caucasian	112	59.6
Indigenous (Canada)^d^	3	1.6
Indigenous (outside of Canada)	0	0.0
Latino/Latina/Latinx/Hispanic	2	1.1
Black (African, Caribbean, Canadian, etc.)	5	2.7
Middle Eastern	22	11.7
East Asian (Chinese, Japanese, Korean, etc.)	19	10.1
Central Asian (Kazakh, Afghan, Tajik, Uzbek, Caucasus, etc.)	1	0.5
South Asian (Indian, Pakistani, Sri Lankan, East Indian from Guyana, etc.)	18	9.6
Southeast Asian (Cambodian, Indonesian, Laotian, Vietnamese, Thai, etc.)	0	0.0
West Asian (Iranian, Iraqi, Persian, etc.)	0	0.0
Other^e^	6	3.2

^a^Academic faculty is defined as a physician whose primary practice is in an often urban tertiary-level hospital and partakes in teaching and research

^b^Community faculty is defined as a physician whose primary practice is in a non-academic hospital with/without a university affiliation

^c^Other options: Gender fluid, non-binary, transgendered, two-spirited, another gender identity

^d^Indigenous includes: First Nation, Inuit, or Metis

^e^Other includes combinations of different ethnicity categories but did not specify which

### Intimidation and harassment

**Fig. 1:**
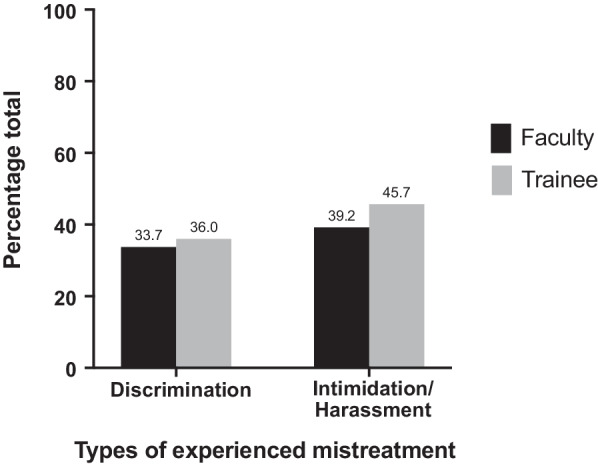
Rates of mistreatment among trainees and faculty

Experiences of intimidation or harassment were reported in 41.8% of all respondents (n = 77/184). The incidence of intimidation or harassment was higher for trainees (45.7%; n = 37/81) than for faculty (39.2%; n = 40/102), corresponding to an absolute difference of 6.5% (95% CI, − 7.9% to 20.9%; P = 0.38) (Fig. 1). When compared to male survey respondents, female respondents reported higher rates of intimidation and harassment (57.0% versus 29.8%), corresponding to an absolute difference of 27.2% (95% CI, 13.2% to 41.2%; P < 0.001). Faculty who were in practice for less than five years reported higher rates of mistreatment (11/21, 52.4%) than faculty who were in practice for greater than five years (34/81, 41.9%), corresponding to an absolute difference of 10.5% (95% CI, − 13.4% to 34.4%; P = 0.39).

**Fig. 2:**
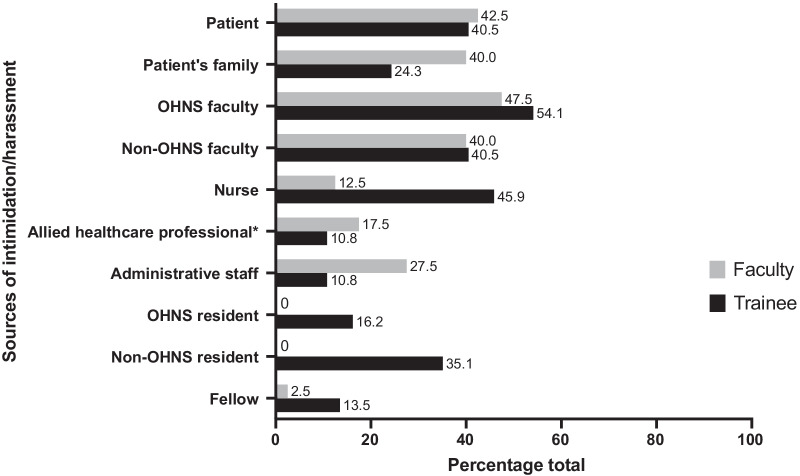
Sources of intimidation or harassment amongst trainees and faculty who experienced mistreatment. *Allied healthcare professional includes nurse practitioners, physiotherapists, speech-language pathologists, etc.

The sources of mistreatment are depicted in Fig. 2. Amongst trainees, the most common sources of intimidation or harassment were from OHNS faculty (54.1%; n = 20/37) and nurses (45.9%; n = 17/37). In comparison, faculty reported fewer incidents of harassment from nurses (n = 5/40; 12.5%). Amongst OHNS faculty, the most common sources of intimidation or harassment were other OHNS faculty (19/40; 47.5%) and patients (17/40; 42.5%). Non-White respondents (faculty and trainees) reported the highest rates of intimidation or harassment from OHNS faculty (n = 20/37; 54.7%) and patients (n = 17/37; 45.9%).

### Discrimination

**Fig. 3:**
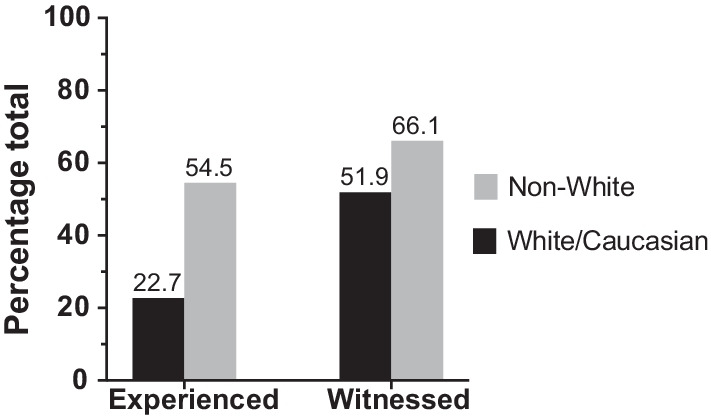
Comparison between rates of experienced versus witnessed discrimination

**Fig. 4:**
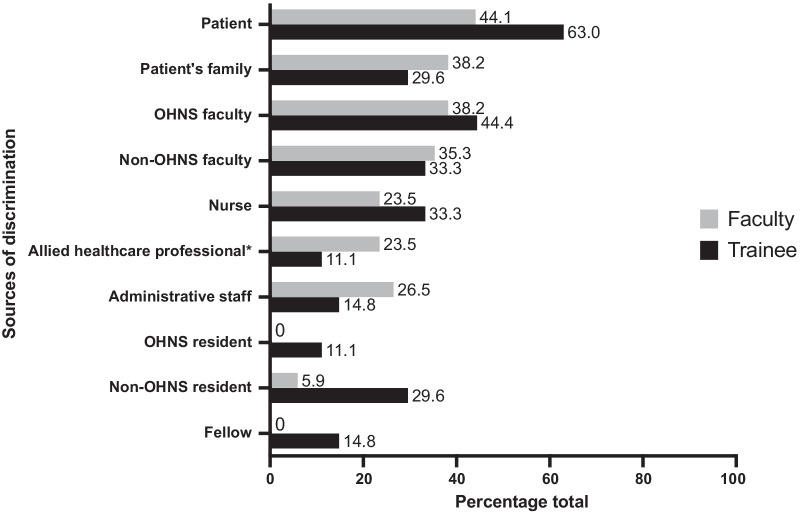
Sources of discrimination amongst trainees and faculty who experienced mistreatment

Discrimination was reported in 34.1% of all survey respondents (n = 61/179). Amongst these, 59.0% (n = 36/61) self-identified as non-White and 41.0% (n = 25/61) self-identified as White/Caucasian. Amongst all survey respondents, White/Caucasian faculty and trainees experienced less discrimination than their non-White colleagues (22.7% versus 49.3%), with an absolute difference of 26.6% (95% CI, 12.6% to 40.6%; P < 0.001). There was also a greater discrepancy between rates of experienced and witnessed discrimination based on ethnicity (Fig. 3). Gender and culture/ethnicity were perceived to be the most common reasons for discrimination (63.2% and 40.4%, respectively). The rates of discrimination as experienced by faculty (n = 34/101; 33.7%) and trainees (n = 27/75; 36.0%) were similar, with an absolute difference of 2.3% (95% CI, − 12.0% to 16.6%; P = 0.75). Patients represented the largest source of discrimination towards trainees (63.0%), followed by OHNS faculty (44.4%) (Fig. 4).

The most commonly reported form of mistreatment was inappropriate verbal comments. When asked about changes in prevalence of mistreatment over time, 60.6% (n = 43/71) of trainees reported no perceived change, in contrast to 31.4% (n = 33/94) of faculty, representing an absolute difference of 29.2% (95% CI, 14.5% to 44.0%; P = 0.001). The majority of faculty (n = 59/94; 62.8%) felt that mistreatment is less prevalent today than early on in their career.

### Mistreatment resources

Most respondents were aware that their affiliated hospitals had mistreatment resources available (60.8%; n = 101/166,). Despite this, only 14.9% of those experiencing mistreatment consulted these resources (n = 17/114). The low utilization was primarily attributed to concerns about retribution (n = 47/72; 65.3%).

Most respondents were unsure if available mistreatment resources were adequate (n = 77/164; 46.9%) or believed them to be inadequate (n = 42/164; 25.6%). Lack of confidentiality was cited as the main reason for inadequacy. When reviewing additional comments submitted by respondents, several individuals noted that there was never any action taken despite their reporting or disclosure of mistreatment which discouraged them and others from reporting further incidents. More than a quarter of respondents who experienced mistreatment further elaborated in the free text section at the end of the survey. The descriptions varied widely in detail and severity of the circumstances surrounding the described episode(s) of mistreatment. Selected anonymized responses are summarized in Table 3.

**Table 3:** Selected anonymized responses from the closing ‘free text’ field of the survey

Respondent	Response
Faculty	I have sought interventions several times from many different sources as listed above for ongoing intimidation, harassment and discrimination—I have come to realize there will never be any action taken to curb the "bad actors"… The deck is constantly stacked against success for me in my experience
Faculty	There is underlying racism and prejudice embedded in the medical world. It is not easily identifiable for those who do not identify with being a visible minority and/or the male gender
Faculty	I would like to see a corrective process for people causing these problems rather than a punitive one. There is a problem that even the suggestions of harassment can destroy a career—this shouldn't be the goal obviously
Faculty	Please broadly disseminate your findings. Medicine is a noble profession, but the culture of medicine which enables intimidation and harassment is toxic
Faculty	I think there have been vast improvements in these issues since I was a resident 30 years ago. In those days I think there was discrimination in the way female residents were thought of vs male residents. There was also a lot of covert homophobia. For the most part I think those areas of discrimination have been eliminated
Trainee	As a woman, myself and my women physician friends have experienced many experiences of discrimination especially from allied health, however you cannot do anything about it otherwise you will become labelled "problematic" as I have seen numerous times
Trainee	When I approached the wellness office at our institution, I was told that action could be taken to deal with the mistreatment I had experienced but that the office was unsure whether this would affect my future employment potential and residency experience and as such recommended that I do not take any action
Trainee	Concerns about confidentiality in reporting are unavoidable no matter how air-tight the reporting process is given how small and interconnected the otolaryngology departments are

## Interpretation

Workplace mistreatment, in the form of intimidation, harassment, and discrimination, remains prevalent within medicine. Various studies have quantified levels of perceived mistreatment amongst learners and faculty between 30 and 85% [5, 10–12]. Physicians work in an immersive environment, often interacting with patients, allied health professionals, nurses, administrative staff, and other physicians. As such, understanding the interpersonal dynamics of mistreatment is imperative when the goal is to cultivate solutions that lead to positive change. Within OHNS, there is a lack of data characterizing the source, nature, and overall prevalence of mistreatment. This survey study aimed to quantify mistreatment and understand how it manifests within OHNS, a crucial first step in addressing this ubiquitous problem.

Canadian OHNS physicians surveyed in our study experienced workplace mistreatment at a mean rate of 47.6%. Both faculty and trainees were affected by mistreatment, but trainees to a greater degree. OHNS faculty were the most common source of intimidation and harassment towards both trainees and other faculty members. Patients and their families were the most common sources of discrimination. Moreover, overall mistreatment was experienced at a greater rate by females—with gender being the most common perceived reason for mistreatment. Other factors associated with mistreatment include non-White ethnicity and junior faculty status.

The hierarchical structure of medicine and a lack of diversity are historical factors that have contributed to mistreatment in the clinical setting [10, 13]. Several respondents alluded to the “toxic” hierarchical culture of medicine as one that allows mistreatment to remain prevalent (Table 3). In recent years, medicine, as a structural entity, is challenging the historical reliance on “dysfunctional” hierarchies and moving towards “functional” hierarchies which rely on an inclusive environment to improve education, learner well-being, and patient safety [10]. Moreover, dysfunctional hierarchies often reinforce an exclusionary culture that may shame those speaking out about mistreatment [7].

Despite a majority of institutions having mistreatment policies and resources available, there was a discrepancy between the number of people experiencing mistreatment and those utilizing the available resources. Overwhelmingly, the principal reason preventing utilization of resources was due to fear of retribution and breach of confidentiality. Given the relatively small size of OHNS departments, it can be inherently challenging to protect anonymity (Table 3). Furthermore, trainees believed that their ability to secure future employment could be compromised, especially considering the largest source of intimidation and harassment was OHNS faculty. Several of the trainees who reported mistreatment and consulted resources described being met with blame or inaction. Hesitancy in accessing program-based resources may be addressed with a third-party ombudsman tasked with addressing mistreatment concerns—such as the newly formed Office of Learner Experience at our institution. Such structural entities that establish mistreatment policies and anonymized reporting mechanisms are a crucial first step. Awareness of these resources, culture change through strong leadership, and accountability are essential to fostering a safe and supportive work and training environment.

Nearly half of all faculty experiencing mistreatment reported it as coming from another faculty member. This finding highlights that mistreatment extends beyond residency and underscores the importance of an accountable support system. There was also a significant portion of mistreatment coming from patients and patients’ families. Though complete prevention is difficult, several mitigation strategies have been suggested to better prepare trainees and faculty when faced with difficult patient encounters. These include formal team debriefing sessions, cultural competency education, awareness of the chain of command for escalation, creation of multidisciplinary task forces focused on education efforts and policy changes, as well as mistreatment surveys, such as the one executed herein, for longitudinal tracking [14, 15].

There are several limitations to this study. Given the need for respondents to reflect on events of the past year, there is an element of recall bias. Moreover, as this survey contained the words “mistreatment” and “discrimination” in the title, it is possible that that those who experienced mistreatment were more likely to answer the survey, therefore overestimating the rates of mistreatment we observed. A second limitation is the low response rate of 39.1%. Although this rate is on par with other national surveys within the profession (20–40%), our results should be interpreted with caution due to participation bias [16–18]. Moreover, the relatively low number of respondents and overall homogeneity in particular demographic categories prohibit more robust statistical analysis, which may have provided some important insights. Finally, as the survey was disseminated by our study team based out of the University of Toronto, there is a possibility of bias towards respondents from the University of Toronto over other institutions in Canada.

## Conclusion

Mistreatment is experienced by both faculty and trainees within OHNS departments across Canada. Trainees face disproportionately more mistreatment overall. Non-White physicians and females also face higher rates of racial and gender discrimination, respectively. Though most institutions had mistreatment resources available, those who experienced mistreatment did not access them mainly due to concerns of confidentiality and retribution.

## Data Availability

The datasets used and/or analysed during the current study are available from the corresponding author on reasonable request.

## Abbreviations

OHNS: Otolaryngology Head and Neck Surgery
VM: Visible minority

## References

[1] Elmore LC, Jeffe DB, Jin L, Awad MM, Turnbull IR (2016). National survey of burnout among US general surgery residents. J Am Coll Surg.

[2] Golub JS, Johns MM, Weiss PS, Ramesh AK, Ossoff RH (2008). Burnout in academic faculty of otolaryngology-head and neck surgery. Laryngoscope.

[3] Golub JS, Weiss PS, Ramesh AK, Ossoff RH, Johns MM (2007). Burnout in residents of otolaryngology-head and neck surgery: a national inquiry into the health of residency training. Acad Med.

[4] Cheng MY, Neves SL, Rainwater J (2020). Exploration of mistreatment and burnout among resident physicians: a cross-specialty observational study. Med Sci Educ.

[5] Hu Y-Y, Ellis RJ, Hewitt DB (2019). Discrimination, abuse, harassment, and burnout in surgical residency training. N Engl J Med.

[6] Wong RL, Sullivan MC, Yeo HL, Roman SA, Bell RH, Sosa JA (2013). Race and surgical residency: results from a national survey of 4339 US general surgery residents. Ann Surg.

[7] Lawlor C, Kawai K, Tracy L, Sobin L, Kenna M (2021). Women in otolaryngology: experiences of being female in the specialty. Laryngoscope.

[8] Statistics Canada Census. 2016 Census. 2016.

[9] Mocanu V, Kuper TM, Marini W (2020). Intersectionality of gender and visible minority status among general surgery residents in Canada. JAMA Surg.

[10] Salehi PP, Jacobs D, Suhail-Sindhu T, Judson BL, Azizzadeh B, Lee YH (2020). Consequences of medical hierarchy on medical students, residents, and medical education in otolaryngology. Otolaryngol Head Neck Surg.

[11] Sheehan KH, Sheehan DV, White K, Leibowitz A, Baldwin DC (1990). A pilot study of medical student 'abuse': student perceptions of mistreatment and misconduct in medical school. JAMA.

[12] Al-Shafaee M, Al-Kaabi Y, Al-Farsi Y (2013). Pilot study on the prevalence of abuse and mistreatment during clinical internship: a cross-sectional study among first year residents in Oman. BMJ Open.

[13] Crowe S, Clarke N, Brugha R (2017). ‘You do not cross them’: hierarchy and emotion in doctors' narratives of power relations in specialist training. Soc Sci Med.

[14] Whitgob EE, Blankenburg RL, Bogetz AL. The discriminatory patient and family: strategies to address discrimination towards trainees. *Acad Med.* 2016;91(11 Association of American Medical Colleges Learn Serve Lead: Proceedings of the 55th Annual Research in Medical Education Sessions):S64–S69. 10.1097/ACM.0000000000001357 27779512

[15] Wheeler DJ, Zapata J, Davis D, Chou C (2019). Twelve tips for responding to microaggressions and overt discrimination: When the patient offends the learner. Med Teach.

[16] Wu V, Manojlovic Kolarski M, Kandel CE, Monteiro E, Chan Y (2021). Current trend of antibiotic prescription and management for peritonsillar abscess: a cross-sectional study. Laryngoscope Investig Otolaryngol.

[17] Kuhnow A, Al-Sayed AA, Taylor B (2022). Routine evaluation of tonsillectomy specimens: a cross-sectional survey of Canadian otolaryngology: head and neck surgeons. J Otolaryngol Head Neck Surg.

[18] Cottrell J, You P, Fung K (2019). Factors influencing the choice of practice location among Canadian otolaryngologists. J Laryngol Otol.

[19] Occupational Health and Safety Act, R.S.O. 1990, c. O.1. In: Ontario Ministry of Labour TaSD, ed2020.

[20] Ontario Human Rights Commission. Policy and guidelines on racism and racial discrimination. In. Toronto: Ministry of Citizenship, Government of Ontario2005.

